# Coronary Sinus Rupture Due to Blunt Cardiac Trauma

**DOI:** 10.1016/j.jaccas.2021.11.024

**Published:** 2022-01-19

**Authors:** Ngoc Tu Vu, Duy Thang Nguyen, Minh Ngoc Le, Duy Gia Nguyen, Quoc Hung Doan

**Affiliations:** a Department of Surgery, Hanoi Medical University, Hanoi, Vietnam; b Department of Cardiovascular and Thoracic Surgery, Hanoi Medical University Hospital, Hanoi, Vietnam

**Keywords:** cardiac rupture, coronary sinus, heart injuries, CPB, cardiopulmonary bypass, CT, computed tomography

## Abstract

We present a very rare case of devastating blunt cardiac trauma with large right atrial rupture, contusion of the right atrioventricular groove, and coronary sinus tear. Surgical repair was successfully performed by urgently establishing cardiopulmonary bypass via the femoral vein and artery simultaneously with a median sternotomy. (**Level of Difficulty: Intermediate.**)

## Central Illustration

**Figure:**
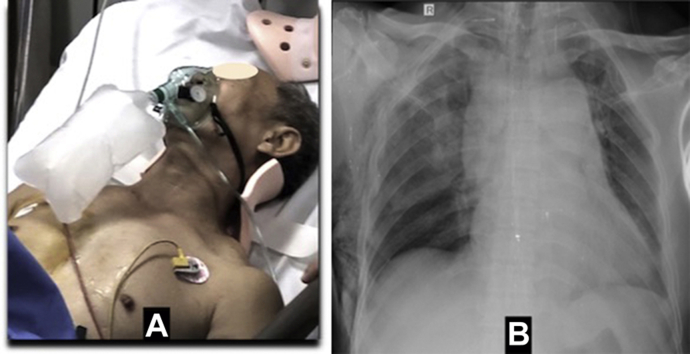

**Figure 1:**
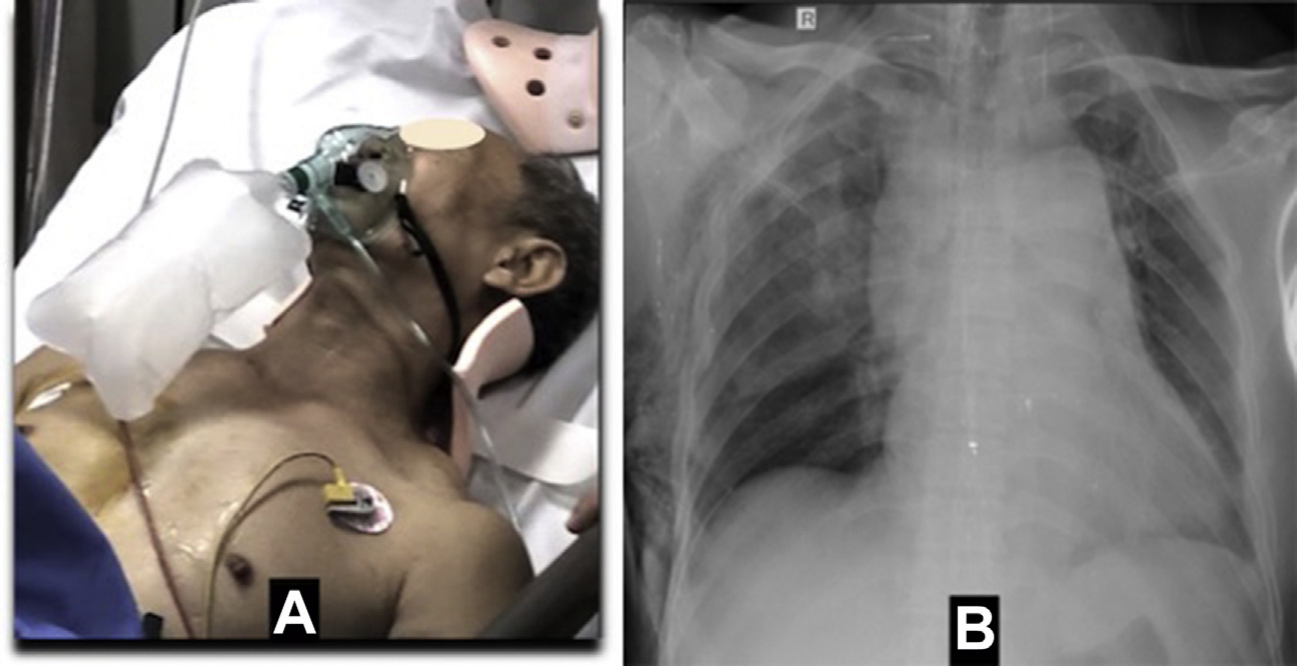
Initial Findings **(A)** Patient with unremarkable thoracic injury. **(B)** Chest radiograph revealing large mediastinum, right subcutaneous emphysema, rib fractures, and pneumothorax.

In June 2021, a 64-year-old man crashed his motorcycle into a vehicle. He was transferred to our hospital after about 1 hour. The primary survey revealed an intact airway. He did not have a traumatic brain injury, but he showed signs of agitation. His initial vital signs were as follows: systolic blood pressure 120/80 mm Hg, pulse 102/min, respiratory rate 29 breaths per minute, and oxygen saturation of 94% with supplemental oxygen at 2 L/min. He had an unremarkable thoracic contusion and anterior flail chest (Figure 1A).

**Learning Objectives**

• To be able to diagnose blunt cardiac trauma quickly in the severe general condition of chest trauma
• To be able to make the best surgical approach for a very complex heart injury

## Differential Diagnosis

The patient's clinical condition was diagnosed as a closed chest injury with symptoms of respiratory failure caused by pneumothorax and anterior flail chest.

## Investigations

**Figure 2:**
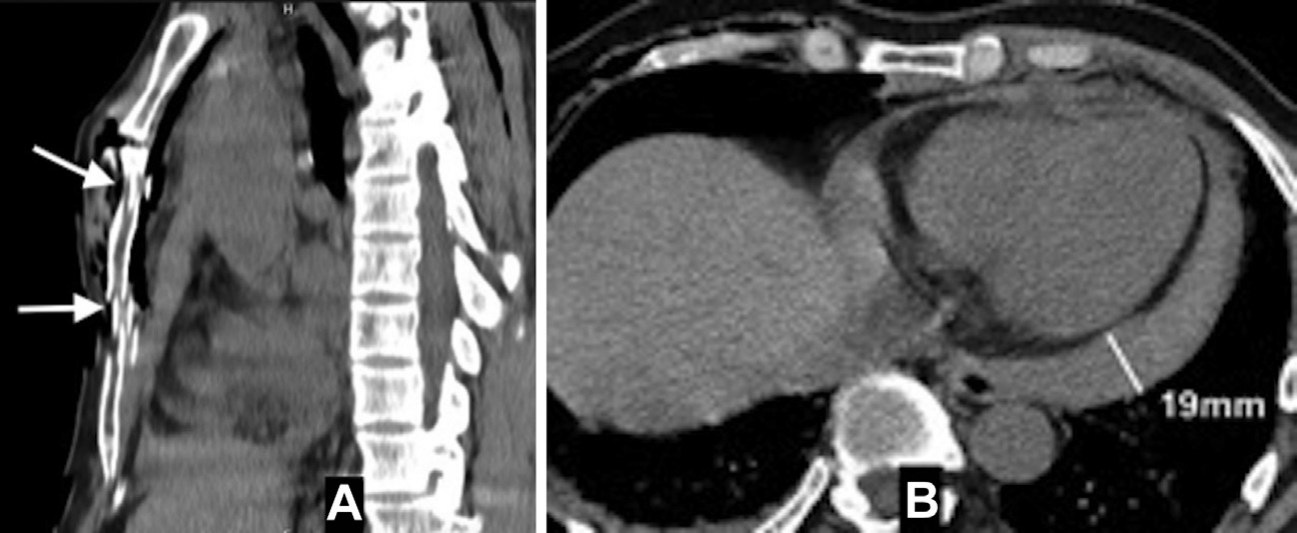
Computed Tomography Findings **(A)** Computed tomographic view demonstrating sternal fracture **(arrowheads)** and **(B)** 19-mm hemopericardium.

A chest radiograph revealed a large mediastinum, right subcutaneous emphysema, rib fractures, and pneumothorax (Figure 1B). An electrocardiogram was normal. The patient underwent emergent drainage of the right side of the chest. After the chest drainage, the patient’s condition was worse, so chest CT scan and bedside transthoracic echocardiography were indicated. The CT demonstrated sternal fracture (Figure 2A) and pericardial hemopericardium (Figure 2B), but no cardiac tamponade was seen by echocardiography (Video 1).

## Management

After echocardiography, his vital signs rapidly deteriorated, and his blood pressure was 70/40 mm Hg, so he was taken to the operating room emergently and expeditiously underwent cardiopulmonary bypass (CPB) via the femoral vein and artery combined with median sternotomy.

**Figure 3:**
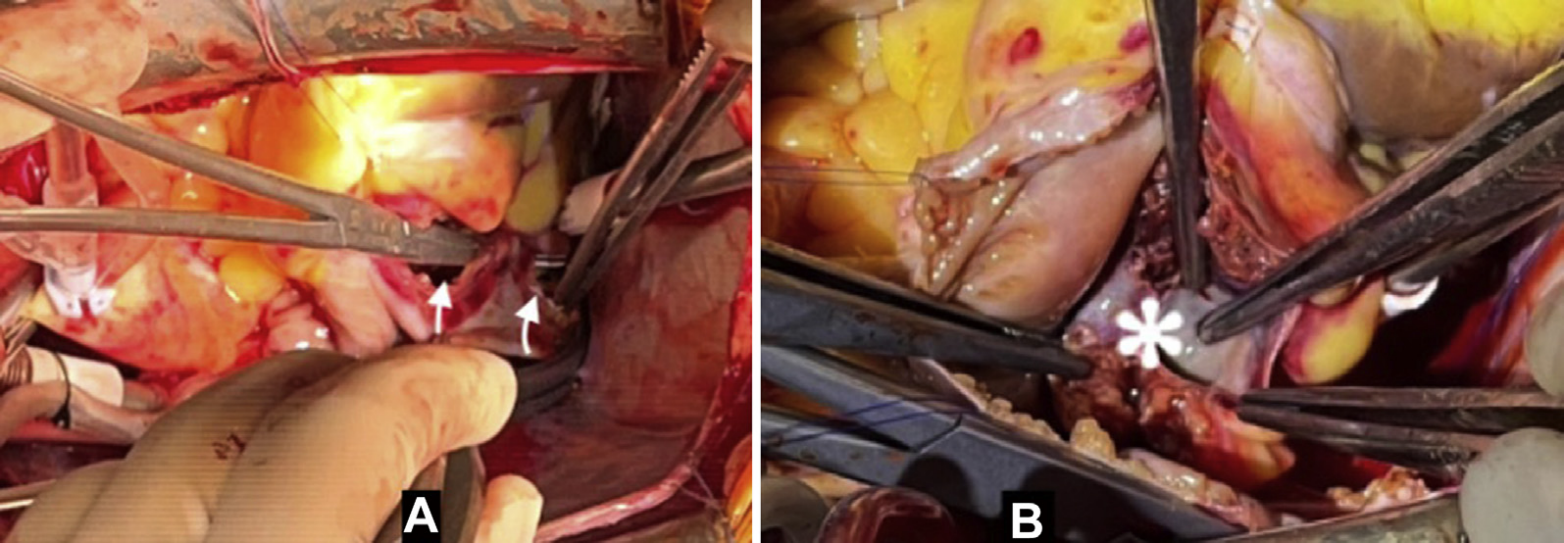
Intraoperative Lesions **(A)** Two large tears on inferior venous-atrial confluence **(arrowheads)**. **(B)** Rupture of the coronary sinus **(asterisk)**.

While the patient was under CPB without an aortic clamp, we found intact pericardium and a large hemopericardium from 2 large tears on the inferior venous-atrial confluence near the atrioventricular groove (Figure 3A).

**Figure 4:**
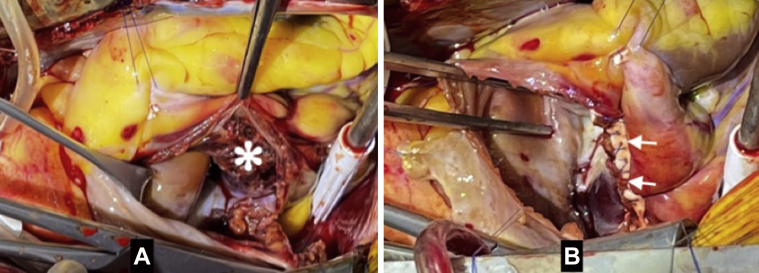
Cardiac Contusion Repair **(A)** Laceration and contusion of the entire right atrioventricular groove **(asterisk)**. **(B)** Repair with double Dacron patch **(arrowheads)**.

The heartbeat was arrested by cold crystalloid cardioplegia. The right atrial chamber was opened, revealing a large contusion and hematoma along almost the entire atrioventricular groove, a tear in the interatrial septum, and rupture of the coronary sinus (Figure 3B). The interatrial septum was sutured directly. The coronary sinus was repaired and reserved. The right atrial wall was reinforced with a double Dacron patch (Figure 4). The injured right atrial wall was sutured into this double patch. The patient was then weaned from CPB normally without a heart block.

Postoperatively, he was in stable hemodynamic condition, and the results of echocardiography were normal. Owing to the sternocostal flail chest and a respiratory infection based on injury and contusion to the lung tissue, he required a long stay in the intensive care unit. He required tracheotomy on the 12th day in the intensive care unit, and he did not need mechanical ventilation on the 27th day. He underwent fixation of the rib, sternum, and left tibia before removal of the tracheotomy on the 44th day and was discharged home on the 52nd hospital day, walking and with normal function and structure on echocardiography.

## Discussion

### Mechanisms and pattern of traumatic heart rupture

Cardiac rupture is the most devastating, and the right side of the heart is most commonly injured.^1^ Rupture of the right atrium after closed chest trauma is due to acute compression of the heart between the sternum and the spine, resulting in increased right-sided heart pressure coincident with phases of the cardiac cycle, leading to high hydrostatic pressure inside the chamber. The same mechanism can injure other right intracardiac structures such as the interventricular septum and the tricuspid valve. Tears at the site of venous-atrial junctions can be explained by the mechanism of sudden deceleration. Anatomically, the ventricle muscle mass is mobile and has most of the heart mass. By contrast, the veins that flow to the atrium are fixed to the pericardial cavity. Therefore, when there is a sudden decrease in acceleration, the ventricle muscle mass continues to move forward or outward while the atrial muscle mass and veins remain in place, leading to tearing of the venous-atrial junction. In our case, because the dislocation of the sternal fracture was unclear and the pericardium was intact, we believe that this multiple heart injury was a combination of 2 main mechanisms: increased intracardiac hydraulic pressure coincident with phases of the cardiac cycle and sudden deceleration at the inferior vena cava and right atrial confluence.

### Surgical treatment

Blunt cardiac ruptures surviving to hospital presentation are exceedingly rare and are associated with a high mortality rate of 89.2%.^1^ Rapid transportation to medical care; accurate, timely diagnosis; and emergent operative intervention are essential for a successful outcome.

The optimal exposure for an isolated cardiac injury is achieved by a median sternotomy. Median sternotomy offers good exposure to all chambers of the heart and also to the great vessels, provides easy access for CPB in complex cases, and can be easily extended into a laparotomy if the need arises.^2^ Because special instruments are needed to divide the sternum, a thoracotomy incision may be preferred in hospitals without open heart surgery teams.^3^

There have been several reports of repair of atrial injury without CPB.^3^ However, it is difficult to be sure whether or not the cardiac rupture in those reports was caused by preoperative atrial injury. In the case series by Namai et al,^4^ 4 of 5 patients underwent CPB. The potential uses of CPB are not limited to cardiac chamber injury. CPB has also been used in the repair of concomitant intracardiac injuries. In our study, the patient had acute hemodynamic decompensation and a risk of cardiac arrest, so he underwent CPB through the femoral vein and artery while the median sternotomy was being performed. CPB with a femoral vein–femoral artery access can provide hemodynamic stability and bleeding control before the pericardium is opened.^4^^,^^5^

Surgical options for the treatment of blunt cardiac trauma include various repair techniques, but the most straightforward option is reasonable in urgent conditions. The cardiac tears can be repaired with simple suture or ligation techniques or patch closure of the large tear.^4^ Pledged or nonpledged polypropylene suture repair had been used in several reports.^6^^,^^7^ Because of the extent of the large laceration and contusion into the atrioventricular groove with the right coronary artery in the present case, resection of this damaged region was not possible. We decided to reserve and reinforce it with a double Dacron patch. Then, the injured right atrial wall was restored into this double patch.

## Follow-Up

Two months post-discharge, the patient came back for a follow-up examination. He could walk independently, was in stable health, and had mild pain at the incision. The patient’s chest x-ray showed his lungs and ribs in good condition, and the size and outline of the heart were normal. His echocardiograms revealed good heart function, and his heart valve structures and cardiac chamber size were normal.

## Conclusions

Blunt cardiac trauma with large right atrial rupture, contusion of right atrioventricular groove, and coronary sinus tear is extremely rare. To save a patient, accurate initial diagnosis and emergency open-heart surgery are the keys to success.

## Funding Support and Author Disclosures

The authors have reported that they have no relationships relevant to the contents of this paper to disclose.

## Appendix

Supplemental Video 1 Echocardiogram revealing hemopericardium and pericardial thrombus (*arrowheads*) but no cardiac tamponade.
